# Eau De Beaute De M. Bargasse

**Published:** 1871-02

**Authors:** 


Eau de Beaute de M. Bargasse.—Eau de Beaute consists of: Bichloride of mercury, 80 centigrms.; camphor, 1 grm.; sulphate of zinc and solution of lead (*liquor plumbi acetici*), of each 4 grms.; rosewater, 250 grms.; and the yelk of one egg. This mixture is regularly in use (according to the *Jahrbuch fur Pharm.*) by Creole ladies for beautifying their skin. A bottle containing this mixture in the above quantity is sold for about five shillings.

